# Differences in the Endophytic Microbiome of Olive Cultivars Infected by *Xylella fastidiosa* across Seasons

**DOI:** 10.3390/pathogens9090723

**Published:** 2020-09-02

**Authors:** Annalisa Giampetruzzi, Paula Baptista, Massimiliano Morelli, Cristina Cameirão, Teresa Lino Neto, Daniela Costa, Giusy D’Attoma, Raied Abou Kubaa, Giuseppe Altamura, Maria Saponari, José Alberto Pereira, Pasquale Saldarelli

**Affiliations:** 1 Dipartimento di Scienze del Suolo, della Pianta e degli Alimenti, Università degli Studi di Bari, 70126 Bari, Italy; annalisa.giampetruzzi@uniba.it; 2 Centro de Investigação de Montanha (CIMO), Campus de Santa Apolónia, Instituto Politécnico de Bragança, 5300-253 Bragança, Portugal; pbaptista@ipb.pt (P.B.); ccameirao@ipb.pt (C.C.); jpereira@ipb.pt (J.A.P.); 3 Consiglio Nazionale delle Ricerche, Istituto per la Protezione Sostenibile delle Piante, Sede Secondaria di Bari, 70126 Bari, Italy; massimiliano.morelli@ipsp.cnr.it (M.M.); giusy.dattoma@ipsp.cnr.it (G.D.); raied.aboukubaa@ipsp.cnr.it (R.A.K.); giuseppe.altamura@ipsp.cnr.it (G.A.); maria.saponari@ipsp.cnr.it (M.S.); 4 Biosystems & Integrative Sciences Institute (BioISI), Plant Functional Biology Center (CBFP), Campus de Gualtar, University of Minho, 4710-057 Braga, Portugal; tlneto@bio.uminho.pt (T.L.N.); daniela.ffc22@gmail.com (D.C.)

**Keywords:** *Xylella fastidiosa*, bacteria, fungi, archaea, 16S/ITS sequencing, shotgun metagenomic sequencing, kalamata, FS17, resistance

## Abstract

The dynamics of *Xylella fastidiosa* infections in the context of the endophytic microbiome was studied in field-grown plants of the susceptible and resistant olive cultivars Kalamata and FS17. Whole metagenome shotgun sequencing (WMSS) coupled with 16S/ITS rRNA gene sequencing was carried out on the same trees at two different stages of the infections: In Spring 2017 when plants were almost symptomless and in Autumn 2018 when the trees of the susceptible cultivar clearly showed desiccations. The progression of the infections detected in both cultivars clearly unraveled that *Xylella* tends to occupy the whole ecological niche and suppresses the diversity of the endophytic microbiome. However, this trend was mitigated in the resistant cultivar FS17, harboring lower population sizes and therefore lower *Xylella* average abundance ratio over total bacteria, and a higher α-diversity. Host cultivar had a negligible effect on the community composition and no clear associations of a single taxon or microbial consortia with the resistance cultivar were found with both sequencing approaches, suggesting that the mechanisms of resistance likely reside on factors that are independent of the microbiome structure. Overall, *Proteobacteria*, *Actinobacteria*, *Firmicutes*, and *Bacteriodetes* dominated the bacterial microbiome while *Ascomycota* and *Basidiomycota* those of Fungi.

## 1. Introduction

*Xylella fastidiosa* is a Gram-negative gamma proteobacterium in the family *Xanthomonodaceae*, of which three main subspecies are described, *multiplex*, *fastidiosa*, and *pauca* [1], all originating from the Americas. The bacterium is a major threat for European and Mediterranean agriculture, being capable of infecting different crop species and to establish itself in different Mediterranean agro-ecosystems, causing in some cases severe diseases [2]. Infections of this polyphagous and xylem-dwelling bacterium may prove asymptomatic in several host species while they can induce severe diseases in crops of agricultural importance. Citrus variegated chlorosis (CVC), affecting fruit size and quality of oranges and Pierce’s Disease (PD), inducing leaf scorching and grapevine decline, are among the most destructive and economically important diseases caused by *Xylella* [3]. The olive quick decline syndrome (OQDS), a novel disease described for the first time in 2013 in southern Italy [4], represents an example of the detrimental impacts associated to this pathogen spreading on the Mediterranean territories and infecting a traditional and widespread species. A hitherto uncharacterized genotype (namely the sequence type ST53) of *X. fastidiosa* subspecies *pauca* was found to be the causal agent of OQDS, which, coupled with abundant populations of the local xylem-feeding insects (primarily the so-called “spittlebugs”), determined an epidemic spread of the pathogen, currently affecting an area of approximately 750,000 ha [2]. The ability to infect up to 595 plant species [5], together with the insect transmission [6] and the lack of effective treatments to cure infected plants [2], make the control of *Xylella* infections very challenging, requiring a compendium of integrated strategies relying on reducing vector population, eliminating sources of infections, and search for resistance traits in the affected species. Proofs of genetic resistance have been found in grape [7,8] and citrus [9,10,11,12] and more recently in olive cultivars Leccino and FS17 [13,14,15,16,17]. Even so, mechanisms underlying differential host responses to *Xylella* infections are still largely unknown. As for PD, a broad consensus indicates that symptoms are the result of the systemic colonization of the bacterium which blocks the xylem vessels and causes a progressive deficit in water transport. In this scenario, anatomical features and abundant bacterial populations have major roles in impairing xylem conductivity as vascular occlusions are caused by the occurrence of bacterial aggregates and by tyloses, which are outgrowths of parenchyma cells of the xylem produced by plants in response to biotic or abiotic stresses [3,18,19].

While genetic [8,20] and/or anatomo-physiological [7,21] studies have contributed to unravel some of the mechanisms that contribute to constraining *Xylella* multiplication and movement in resistant grapevines, very little is known about the relationships of *X. fastidiosa* with all other microorganisms inhabiting the xylem vessels and their potential role in limiting infections, i.e., contributing to modulate the response in resistant phenotypes. In this framework, we studied the dynamics of *Xylella* plant colonization in relation to the whole olive microbiome to shed light on the complex network of interactions occurring among microorganisms inhabiting the same niche, the xylem vessels.

Strategies to study the plant microbiome compositions rely on the isolation and identification of cultivable microorganisms (cultivation-dependent), or massive sequencing (cultivation-independent) [22,23,24]. The majority of the currently available studies rely on the analysis of next generation sequencing (NGS) datasets from 16S ribosomal RNA gene (16S rRNA) sequences from bacteria or fungi (internal transcribed spacer, ITS) [25]. However, information on the whole microbiome can be also obtained through whole metagenome shotgun sequencing (WMSS) [26,27], a strategy that allows gathering microbial data at very high depth. A combination of both cultivation-dependent and independent approaches can be effectively exploited for identifying beneficial microorganisms or consortia potentially antagonizing known plant pathogens to be used as biocontrol agents.

Studies of the microbial endophytes were reported in *Xylella* pathosystems from citrus, grapevine, and more recently, from olive. Several authors [28,29,30,31,32] described differences in the endophyte populations of asymptomatic and symptomatic citrus plants affected by CVC and proposed that the development of symptoms is the result of an unbalanced ratio among *Methylobacterium* and *Curtobacterium* species, and *X. fastidiosa*. In particular, *Curtobacterium flaccumfaciens* was found to inhibit the growth of *X. fastidiosa* subspecies *pauca* in vitro and to prevent or reduce the symptoms in *Catharanthus roseus* plants when it was co-inoculated with *Xylella* [32]. Similarly, a citrus-isolated strain of *Methylobacterium mesophilicum* inhibited the growth of *Xylella* in vitro and reduced its population in *C. roseus* plants [31]. Cultivation-dependent or independent approaches were similarly used to characterize the grapevine-associated endophyte microbiome inhabiting debarked cane tissues or sap [33,34]. *Proteobacteria* and *Ascomycota* were found to be predominant while the bacterium *Pseudomonas fluorescens* and the fungus *Achromobacter xyloxidans* were found to be inversely correlated with the *X. fastidiosa* subspecies *fastidiosa* populations in grapevine escaping the Pierce’s Disease [34].

The dynamics of the microbial communities in the xylem sap of grapevines under high Pierce’s Disease pressure was described in three different phenological stages over two growing seasons [33]. This study led to the identification of the grapevine core bacterial and fungal microbiomes of plants showing mild, moderate, or severe Pierce’s Disease symptoms. Furthermore, the microbial diversity richness in the grape xylem sap was highest during bloom while the disease condition, as well as the phenological stage, shaped the microbial communities.

The majority of studies on the olive microbiome based on cultivation-dependent or independent sequencing, independently of *Xylella* infections, targeted the rhizosphere compartment [35,36,37,38] while they are limited for endophytes of aboveground tissues. Using 16S rRNA gene amplicon sequencing, Müller et al. [39] found that the bacterial endophyte communities from leaves and boughs of wild and cultivated olives were largely shaped by the plant genotype and correlated with the geographic origin. Interestingly, these authors detected a high proportion of Archaea, whose ecological significance remains elusive. Similar to the finding from Müller et al. [39], a predominance of *Proteobacteria*, *Firmicutes*, *Bacteriodetes*, and *Actinobacteria* phyla were found by Fausto et al. [40] in the olive xylem sap. However, differently from Müller et al. [39], in their 16S rRNA gene analysis, no traces of Archaea were found in the xylem sap while these microorganisms were present with low abundances in leaves and soil fractions. A comparison between cultivation-dependent and independent approaches to study the xylem microbiome of olive cultivars Picual, Arbequina, and Acebuche [41] showed that the main factor shaping the xylem-inhabiting microbiome was the olive genotype. Interestingly minor variations in the microbiome composition were detected between the xylem-sap (recovered using the Scholander pressure chamber) and the whole homogenized xylem tissue. A large fraction of bacteria were only detected by culturing (58.8%) and not by amplicon sequencing (16S rRNA gene NGS).

Studies of fungal endophyte communities in olive were mainly performed in aboveground organs by using cultivation-dependent methods [42,43,44,45], while metabarcoding analysis was less frequently used. All these studies indicated *Ascomycota* as the most abundant fungal endophytes in olive leaves, twigs, and fruits. Different factors have been shown to shape olive-associated fungal endophytic composition, including host genotype (at cultivar level), plant organ, seasonality, and presence of pathogens [42,44,45].

Because of OQDS novelty, understanding the pathogen-host interactions and the epidemiology of the infections in the affected area became crucial to develop effective containment measures. A major finding of the studies to contrast the OQDS epidemic in olives was the discovery of olive cultivars showing resistance towards *X. fastidiosa* subspecies *pauca* ST53, namely FS17 and Leccino, as opposed to the susceptible Ogliarola salentina, Cellina di Nardò, and Kalamata [13,14]. Both resistant cultivars were found harboring lower bacterial population sizes and showed less severe symptoms, as compared to Ogliarola salentina and Kalamata [13,14]. Recently, Vergine et al. [46] explored the potential role of microbial endophytes in protecting olive cultivar Leccino from the OQDS, in comparison with Cellina di Nardò. Interestingly, they observed a drastic dysbiosis in response to *X. fastidiosa* infection in Cellina di Nardò, while Leccino maintained microbial communities more stable, and with higher diversity than Cellina di Nardò, in both infected and uninfected plants.

**Figure 1:**
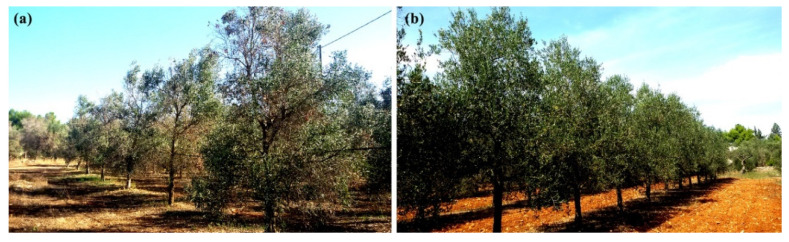
Effect of *X. fastidiosa* infection on olive trees sampled for the aims of this study, in the Apulian outbreak area (Sannicola, Lecce, Italy), as observed in November 2018. Desiccations on the canopy of the susceptible cultivar Kalamata (**a**) were very evident, while the resistant cultivar FS17 (**b**) still appeared without or with very mild symptoms.

In the present work, we studied the microbiomes of 15 years-old trees of the cultivars FS17 and Kalamata, co-cultivated in the same orchard located in the core outbreak area of Apulia, in southern Italy, by using WMSS and 16S/ITS rRNA gene sequencing. Trees were run under the same agricultural practices and subjected to the same environmental conditions. Tissues were sampled in two seasons from plants being initially, during 2017, symptomless and showing, during 2018, advanced or limited symptoms in the cultivars Kalamata and FS17, respectively (Figure 1). The analysis aimed to investigate the changes in the olive xylem microbiome upon *Xylella* infection and to assess whether correlations exist between the composition of the xylem microbiome and the differential phenotypical responses of the two cultivars to *X. fastidiosa* infections. Based on the gathered information, a final goal is to identify bacterial/fungal microbes or consortia associated with the resistant phenotypes to be exploited as potential biocontrol agents.

## 2. Results

To describe the xylem microbiome of olive trees showing differential response to *X. fastidiosa*, a total of 72 libraries were successfully sequenced, respectively, 24 by WMSS (i.e., six samples per two cultivars per two seasons) and the same number by amplicon sequencing of the 16S/ITS rRNA gene.

### 2.1. Description of the Microbiome by Whole Metagenome Shotgun Sequencing (WMSS)

Initial attempts to classify the WMSS data using Kraken, with default *k*-mer size and databases, proved to be unsuccessful in the correct reads assignment, with the majority of those associated to the fungal kingdom, corresponding to plant sequences, as assessed by BLASTn analysis (not shown). We, therefore, re-mapped the sequenced reads with Kraken 2 using a custom database, built using the *kraken2-build* option, from nucleotide sequences of archaeal, bacterial, viral, fungal, and plant complete genomes within the NCBI Reference Sequence (RefSeq) datasets.

Library sizes from April 2017 sampling, ranged between 38,656,227 and 54,871,547 raw reads, of which 97.5% to 98.5% were classified by Kraken 2 as belonging to plant, bacteria, fungi, archaea, and viruses. Similarly, library sizes from trees sampled in November 2018 ranged between 25,255,482 and 48,745,550, of which 97.6% to 98.2% were classified by Kraken 2 as belonging to plant, bacteria, fungi, archaea and viruses (Table 1). A fraction from 1.42% to 2.41% was not classified by Kraken 2 among the 24 libraries. Kraken 2-classified reads were then parsed with MEGAN that assigned 24,597,096–51,202,008 reads (98.31–99.92% of the total, Table 1) to the plant kingdom.

Given the different sizes of the libraries, microbial reads (i.e., bacteria, fungi, archaea and viruses) were normalized according to Regalado et al. [27], by using plant reads as internal spike-in to which microbial reads are referred to. Briefly, normalization takes into account either the average size of all plant reads or the relative abundance of each microbial taxon in the original library. Normalized microbial reads ranged from 38,374 to 1,198,439, whose major fraction was represented by bacteria that, in all libraries were 88.91–99.15% of all microbes (total microbes, Table 1, followed by fungi (0.48–6.40%), viruses (0.21–3.21%), and a(0.11–2.09%) (Table 1). In addition, reads from *Cyanobacteria* were also eliminated as they were found corresponding to rDNA from chloroplasts by BLASTn analysis (data not shown).

Taxa were further filtered by MicrobiomeAnalyst to eliminate those that could be artifacts (i.e., appearing in only one sample) and those having an identical value (i.e., 0) across all samples. Only taxa having a 20% prevalence with a minimum of 10, 50, 10, and 10 reads for Archaea, Bacteria, Fungi and Viruses, respectively, were retained. All libraries were further normalized according to centered log-ratio (clr) transformation. Rarefaction curves of all data reached the plateau and Good’s coverage estimation averaged 99.74%, 98.34%, and 100% (Table S1) for Bacteria, Archaea, and Fungi, respectively, indicating that the majority of diversity was captured with the sequencing effort. However, this result was not achieved for Viruses (see below).

**Figure 2:**
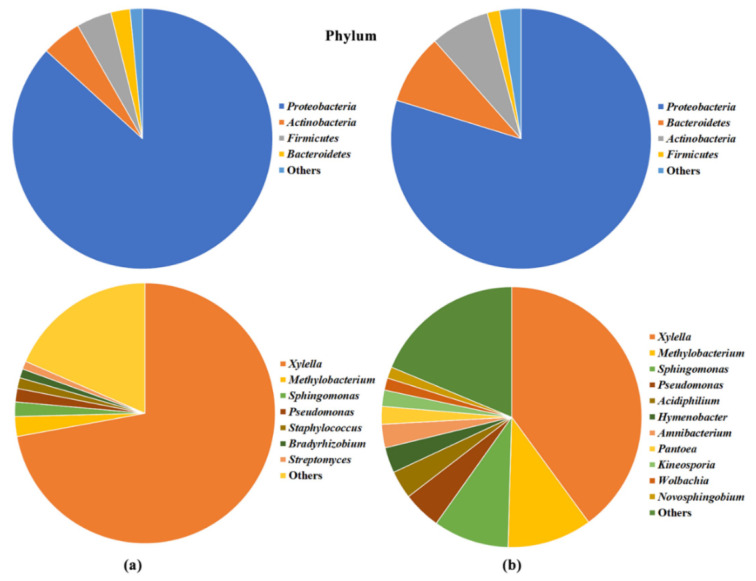
Pie chart representations of the bacteria by (**a**) whole metagenome shotgun sequencing (WMSS) and (**b**) 16S rRNA gene sequencing, in all FS17 and Kalamata olive trees at phylum and genus level. Only taxa with an abundance greater than 1% are reported, while those below this threshold are grouped in the category “Others.”.

**Figure 3:**
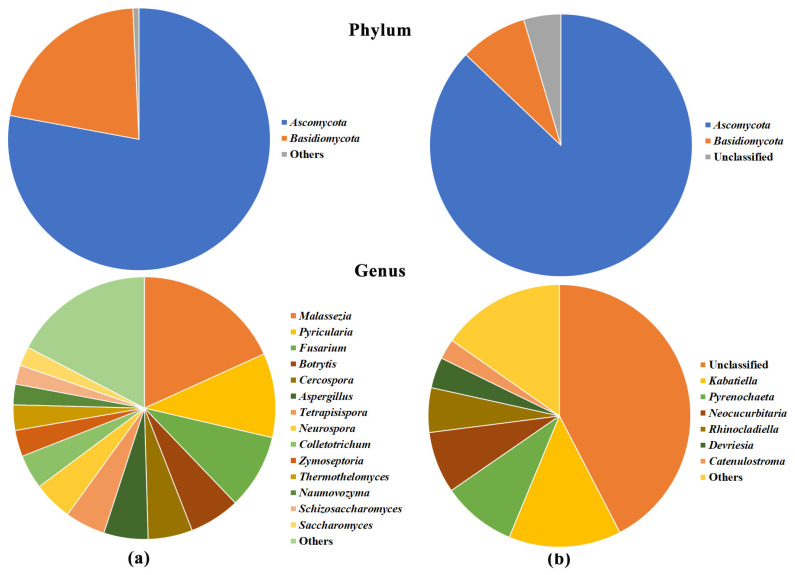
Pie chart representations of the fungal community by (**a**) WMSS and (**b**) ITS1 rRNA gene sequencing in all FS17 and Kalamata olive trees at phylum and genus level. Only taxa with an abundance greater than 2% are reported, while those below this threshold are grouped in the category “Others.”.

**Figure 4:**
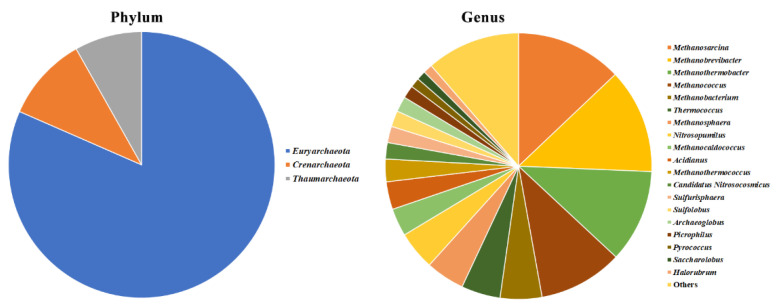
Pie chart representations of the archaeal community by WMSS sequencing in all FS17 and Kalamata olives at phylum and genus level. Only taxa with an abundance greater than 1% are reported, while those below this threshold are grouped in the category “Others.”.

After data filtering and normalization, 12 phyla, 23 classes, 62 orders, 115 families, and 225 bacterial genera (Figure 2a and File S1); three phyla, 10 classes, 13 orders, 19 families, and 29 fungal genera (Figure 3a and File S2); three phyla, 11 classes, 16 orders, 20 families and 34 archaeal genera (Figure 4 and File S3); and 15 viral genera (File S4) were classified. Kraken 2 classification of virus-associated reads was only referred to viruses having a DNA genome and was limited to the genus level, as many taxa had been classified as unassigned. Because of these limited and partial information viruses were not further analyzed.

*Proteobacteria* (86.8%) largely dominated the Bacteria kingdom, while *Actinobacteria* (4.9%), *Firmicutes* (4.4%), *Bacteroidetes* (2.4%), *Tenericutes* (0.7%), *Fusobacteria* (0.4%), and *Spirochaetes* (0.3%) phyla, were limitedly represented (Figure 2a). *Xylella* genus occupied 72.1% of the whole endophytic microbiome, followed by *Methylobacterium* (2.5%), *Sphingomonas* (1.8%), *Pseudomonas* (1.7%), *Staphylococcus* (1.3%), *Bradyrhizobium* (1.1%), *Streptomyces* (1.0%), *Clostridium* (0.9%), and *Friedmanniella* (0.8%).

*Ascomycota* was the major (77.9%) fungal phylum with *Basidiomycota* and *Microsporidia* accounting for 21.4% and 0.7% of total reads, respectively (Figure 3a). At genus level, *Malassezia* (18.2%), *Pyricularia* (10.4%), and *Fusarium* (9.2%) were the most represented, followed by *Botrytis* (6.2%), *Cercospora* (5.5%), *Aspergillus* (5.5%), *Tetrapisispora* (5.0%), *Neurospora* (4.8%), *Colletotrichum* (4.2%), and *Zymoseptoria* (3.2%).

Among the 10 largely represented Archaea genera were *Methanosarcina* (12.9%), *Methanobrevibacter* (12.7%), *Methanothermobacter* (11.3%), *Methanococcus* (10.2%), *Methanobacterium* (5.1%), *Thermococcus* (4.7%), *Methanosphaera* (4.7%), *Nitrosopumilus* (4.7%), *Methanocaldococcus* (3.4%), and *Acidianus* (3.4%), in the majority belonging to the major phylum *Euryarchaeota* (80.1%), followed by *Crenarchaeota* (11.2%) and *Thaumarchaeota* (8.7%) (Figure 4). Plant begomoviruses having a DNA genome were largely the most represented, covering 72% of the viral taxon microbiome (File S4).

These initial filtering and clr normalization were used for all successive studies regarding bacterial, fungal, and archaeal microbiomes.

### 2.2. Description of the Microbiome by 16S and ITS1 rRNA Gene Sequencing

Approximately 99% of a total of 2,056,937 quality-filtered bacterial reads (98.2% of the total reads) were plant-derived sequences (i.e., mitochondrial and plastidial DNA) (Table 2). Whereas in the fungal sequence datasets, no plant sequences were retrieved, in contrast, the percentage of unclassified reads was lower in bacterial (1.8% of the total) than in fungal datasets (27.2%). After removing the operational taxonomic units (OTUs) with low abundance (i.e., less than five or 10 reads for Bacteria and Fungi, respectively), it was observed that there was a larger consortium of Fungi associated with olive tree xylem (535 OTUs, 92 genera, 70 families, 42 orders, 16 classes, and two phyla) than of Bacteria (348 OTUs, 44 genera, 38 families, 29 orders, 16 classes, and 10 phyla), and most of the relatively dominant members within the microbial communities were Fungi (1,756,830 fungal reads), accounting for 99.7% of the total reads. Bacterial communities were predominantly composed by *Proteobacteria* (79.8% of the total bacterial reads), *Bacteroidetes* (8.7%), and *Actinobacteria* (7.3%) phyla (Figure 2b), that translates, at the genus level, with the 39.9% abundance of *Xylella*, followed by *Methylobacterium* (10.5%), *Sphingomonas* (9.4%), *Pseudomonas* (4.7%), *Acidiphilium* (3.5%), *Hymenobacter* (3.2%), *Amnibacterium* (2.9%), *Pantoea* (2.2%), and *Kineosporia* (2.0%), as the most represented taxa. The xylem-inhabiting fungal communities were predominantly dominated by members belonging to *Ascomycota* (87.1% of the total fungal reads) and *Basidiomycota* (8.3%) phyla, while 4.6% was unclassified (Figure 3b). Conversely, 42.4% of the reads could not be assigned to a genus, while most represented genera were *Kabatiella* (13.9%), *Pyrenochaeta* (9.1%), *Neococurbitaria* (7.6%), and *Rhinocladiella* (5.5%).

Comparing the bacterial microbiome composition from the WMSS and the 16S rRNA gene approaches, a strong concordance was found at phylum, class, and order levels (Pearson’s r = 0.99, 0.87, and 0.93, respectively) considering the 10 dominant taxa (File S1). Conversely, a more distant agreement was found among fungal microbiome compositions obtained with both approaches, likely because of the very limited number of sequences (107,821) classified following the metagenome approach (Table 1) comparing to those (1,756,830) obtained with the amplicon sequencing (Table 2; File S2).

### 2.3. Olive Xylem Microbiome Composition by WMSS Analysis

The normalization of the different WMSS libraries against the plant reads allowed to estimate the *Xylella* abundances within each bacterial microbiome and to make comparative analysis among different trees, without any PCR-biases which conversely may occur with 16SrRNA gene approach. The minimum number of normalized reads detected in the sequenced libraries (Table 1) and classified as *Xylella* by Kraken 2 corresponded to 43 in the tree “Kal1-55” and were confirmed by BLASTn analysis. The qPCR assay of the same DNA template yielded a negative result, suggesting a possible higher sensitivity of the high-throughput sequencing technology compared to qPCR.

It could be observed that during both sampling periods the proportion of *Xylella* vs. the total bacterial reads was always lower in trees of cultivar FS17 than in Kalamata and it increased in both cultivars as infections progressed in time (i.e., 2.32% FS17 vs. 8.69% Kalamata in Spring 2017 and 31.48% FS17 vs. 52.67% Kalamata in Autumn 2018) (Table 1). Data from WMSS showed that all selected trees contained *Xylella*-derived sequences, although at the start of the study in most of the trees the bacterium was close to the threshold of detectability by qPCR (i.e., Cq > 30). The *Xylella*/Bacteria relative read abundance significantly correlated (r = 0.63, *p* < 0.001) with *Xylella* population size (CFU/mL) estimated by qPCR detection (Figure S1). Indeed, a one-way ANOVA comparison of the average estimated sizes of *X. fastidiosa* populations (Figure S2) revealed that significant differences existed among plants of the two cultivars when considered in the two sampling periods (Table S2). Moreover, the Tukey’s HSD post-hoc pairwise comparison showed that *X. fastidiosa* populations: (1) Were similar between the two cultivars at the start of the experiment; (2) did not significantly change in FS17 between the two years and the two sampling periods (compare FS17 April 2017 vs. November 2018); (3) increased more rapidly in Kalamata (compare Kalamata April 2017 vs. November 2018).

Low rates of *Xylella*-reads were found in cultivar FS17, with only one sample yielding values higher than 50%, while values higher than 50% were frequent in the libraries prepared from the trees of the cultivar Kalamata (Table 1), for which one of the libraries exhibited value higher than 90% (Kal2-53). In detail, and considering every single plant throughout the two sampling seasons, only in one FS17 tree, FS2-43, *Xylella* relative abundance represented more than 50% of the total Bacteria, while this occurred in four olives (Kal1-89, Kal2-53, Kal2-55, and Kal2-89) of the cultivar Kalamata (Table 1). These high *Xylella* relative abundances, which particularly in the cultivar Kalamata reached even 90.05% of total Bacteria (Kal2-53), suggest that this bacterium tends to occupy the whole bacterial niche. A finding that is demonstrated by the existence of a linear correlation (R^2^ coefficient: 0.92) among *Xylella* and total Bacteria (Figure S2 and Table 1) reads, showing that when total bacterial reads increase in a sample, the increase was mainly due to *Xylella* reads.

Based on the existence of a linear correlation between *Xylella* average population size (CFU/mL) and *Xylella*/Bacteria relative abundance (Figure S3) an arbitrary threshold, corresponding to 5% of *Xylella*-reads over the whole Bacteria, was selected and used to categorize the samples with high (FS1-43, FS2-10, FS2-18, FS2-43, FS2-45, Kal1-89, Kal2-89, Kal2-53, Kal2-54, Kal2-55, Kal2-65) or low (FS1-1, FS1-3, FS1-10, FS1-18, FS1-45, FS2-1, FS2-3, Kal1-53, Kal1-54, Kal1-55, Kal1-57, Kal1-65, Kal2-57) *Xylella* populations. The threshold was selected based on the occurrence of at least one of the two criteria, 5% WMSS *Xylella* abundance and/or population size higher than 5 Log CFU/mL (1.0E + 05, Table 1). This distinction/condition has been used in all following analyses.

**Figure 5:**
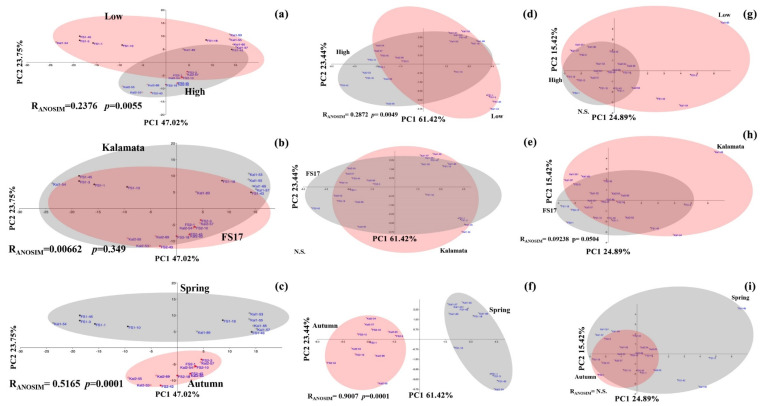
Principal component analysis and ANOSIM test using clr-normalized data of bacterial (**a**–**c**), fungal (**d**–**f**), or archaeal (**g**–**i**) microbiomes from all FS17 and Kalamata plants. Clustering is according to *Xylella* abundance (high vs. low) (**a**,**d**,**g**), cultivar (FS17 vs. Kalamata) (**b**,**e**,**h**), and season (Spring vs. Autumn) (**c**,**f**,**i**). ANOSIM test showed the R-statistic (R) and the statistical significance (*p*). Olives sampled in Spring and Autumn are respectively in black and red colors, while dots and diamonds indicate FS17 and Kalamata olives, respectively. N.S.: Not significant.

Analysis of similarities (ANOSIM) was performed on bacterial, fungal and archaeal communities inhabiting FS17 and Kalamata xylem, to assess the statistical significance of sample groupings and evaluate factors having a major role in shaping the microbiomes. Principal component analysis (PCA) and ANOSIM significantly (R = 0.5165, *p* = 0.0001. Figure 5c; Table S3) separated olives sampled in Spring 2017 from those sampled in Autumn 2018, indicating that season was the main factor shaping bacterial communities either considering all olives or separately those of the two cultivars (R = 0.5481, *p* = 0.0052 in cultivar FS17; R = 0.6111, *p* = 0.002 in cultivar Kalamata). A further factor driving bacterial community composition was *Xylella* that significantly distinguishes trees with low and high abundance (R = 0.2376, *p* = 0.0055; Table S3), although its effect was different when cultivars were separately considered. Indeed, a significant separation was observed in cultivar Kalamata (R = 0.4611, *p* = 0.0081; Table S3) while it was not (R = 0.2424, *p* = 0.0774; Table S3) in cultivar FS17, indicating that microbiomes of the latter cultivar are not heavily affected by the presence of Xylella. No significant differences occurred among olives sampled in Spring 2017 either considering *Xylella* abundance or between the two cultivars (Table S3). While *Xylella* makes a significant difference among samples analyzed during Autumn 2018 (R = 0.3504, *p* = 0.0155; Table S3), this was not related to the cultivar (R = 0.1093, *p* = 0.1541; Table S3).

The exclusion of *Xylella* from the data did not change the overall clustering of samples in PCA analysis and significance in ANOSIM (not shown). Collectively, the analysis of these data showed that *Xylella* abundance and season played a major role in driving the olive bacterial microbiome in both cultivars and *Xylella* shaped mainly the microbiome of the susceptible cultivar Kalamata, while it did not significantly affect that of cultivar FS17. Moreover, our analysis was not biased by the inclusion of the data from *Xylella*, although this taxon occupies the majority of the bacterial niche in some plants indicating that clr transformation efficiently decreases the influence of highly abundant microorganisms. To reduce the bias of highly abundant bacterial taxa an alternative strategy for data normalization was attempted and was based on the fourth root transformation of the reads. However, the fourth root performed worse than clr transformation, as *Xylella* effect on shaping the microbiomes PCA distribution was very significant (not shown). Indeed, excluding *Xylella* from the PCA analysis, a significant separation according to the season of sampling and *Xylella* abundance were obtained (not shown), as observed with clr-transformed data.

A major factor distinguishing the overall fungal microbiome was the period of sampling (Figure 5f; Table S4). A very high ANOSIM R-value supported this distinction by very low *p*-values (R = 0.9007, *p* = 0.0001) for both cultivars, as well as for FS17 (R = 0.9611, *p* = 0.0021) and Kalamata (R = 0.8185, *p* = 0.0019) separately. This indicates that fungal communities are strictly related to the seasonal physiological state of olives and environmental conditions. Moreover, *Xylella* significantly affected the fungal community of all plants (Figure 5d; R = 0.2872, *p* = 0.0049, Table S4) and this effect occurred significantly on the FS17 microbiomes (R = 0.3382, *p* = 0.025; Table S4) while moderately (R = 0.2296, *p* = 0.074; Table S4) on those of the cultivar Kalamata. No significant differences were found among microbiomes of the two cultivars either considering all olives or those having high or low *Xylella* abundances and plants sampled in Spring 2017 or Autumn 2018 (Figure 5e; Table S4).

Archaeal communities were not substantially affected by the three considered factors: Cultivar, season, and *Xylella*. Although significant differences were observed among all plants belonging to the two cultivars (Figure 5h; R = 0.09238, *p* = 0.0504; Table S5) these were very small as can be inferred by the low value of the R-value. Only a slightly significant difference was determined among Kalamata communities sampled in the two seasons (R = 0.2185, *p* = 0.035; Table S5). While no significant differences were observed among FS17 and Kalamata microbiomes sampled in the two periods (Figure 5i and Table S5), neither among those having low or high *Xylella* abundances (Figure 5g and Table S5). This lack of separation was reflected by the lack of specific Archaea genera driving the microbiomes (not shown), which further confirmed the independence of Archaea from any of the three variables (cultivar, season, and *Xylella*) considered. Because of these findings, no further analyses were carried out with Archaea.

**Figure 6:**
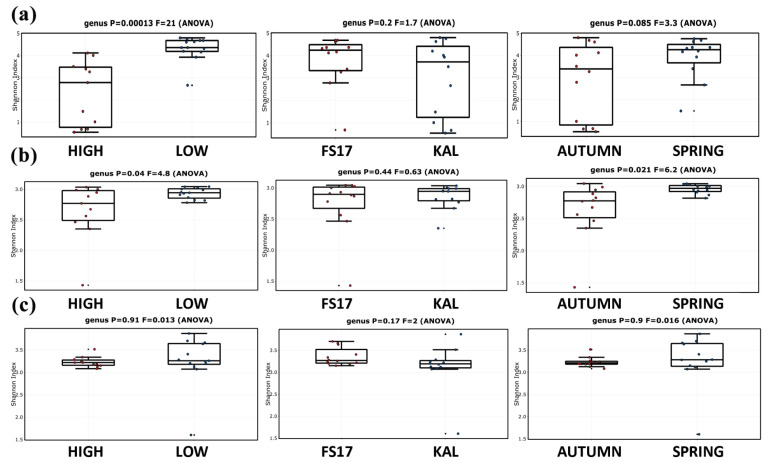
Alpha diversity (Shannon diversity index) of (**a**) bacterial, (**b**) fungal, and (**c**) archaeal microbiomes from all sampled olives. Diversities were compared between plants harboring high or low *Xylella* infections, cultivars, or season of sampling. Boxplots depict medians (central horizontal lines), the interquartile ranges (boxes), and 95% confidence intervals (whiskers). ANOVA test showed the F-value (F) and the statistical significance (*p* < 0.05).

Considering both periods of sampling, the alpha diversity of bacteria (Figure 6a), fungi (Figure 6b), and archaea (Figure 6c) FS17 microbiomes was higher than that of Kalamata, although these differences were not significant. Similarly, a lower diversity was found in bacterial and fungal microbiomes of plants sampled during Autumn 2018, as compared to those from Spring 2017, although this was significant only for Fungi. Conversely, intra-plants diversity significantly dropped in bacterial microbiomes of olives containing high *Xylella* populations, independently of the cultivars, as could be expected in plants were the bacterium tends to occupy the whole ecological niche (Figure 6a). A lower and significant diversity was also found in fungal microbiomes of plants with high *Xylella* abundance, while it was not significant for Archaea, although following the same trend (Figure 5c and Figure 6b).

### 2.4. Olive Xylem Microbiome Composition by 16S and ITS1 rRNA Gene Analysis

**Figure 7:**
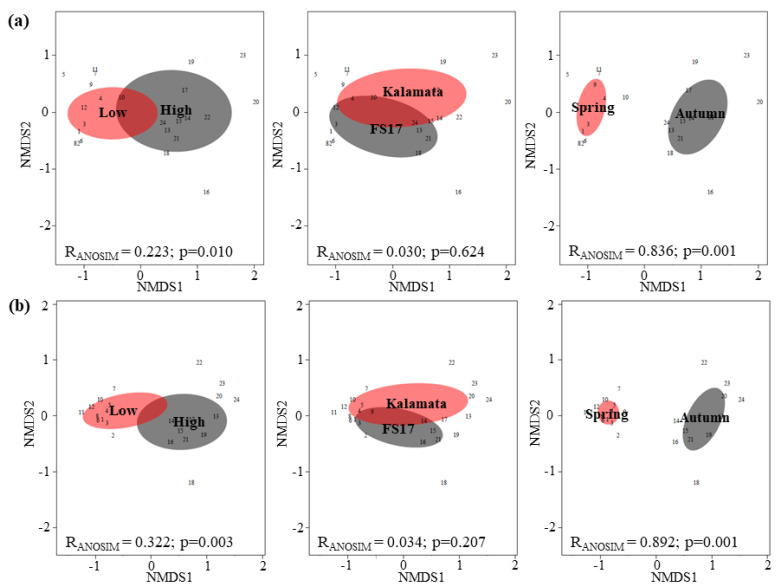
Nonmetric multidimensional scaling (NMDS) plots and ANOSIM test for the (**a**) bacterial and (**b**) fungal assemblages in the xylem of olive trees due to different *Xylella* abundance (high vs. low), host cultivar (FS17 vs. Kalamata), and season (Spring vs. Autumn). Bray–Curtis coefficient was used as a measure of similarity between populations and Kruskal’s stress values obtained for bacteria and fungi were 0.097 and 0.087, respectively. ANOSIM test showed the R-statistic (R) and the statistical significance (*p*).

Nonmetric multidimensional scaling (NMDS) plotting was carried out using 16S rRNA gene data to describe similarities/differences among microbiomes from FS17 and Kalamata trees and the significance of clustering was tested by ANOSIM and PERMANOVA analysis. The NMDS plots and ANOSIM analysis showed that the whole bacterial (Figure 7a) and fungal (Figure 7b) communities composition differ significantly between seasons (Spring vs. Autumn; R = 0.836, *p* = 0.001 for Bacteria; R = 0.892, *p* = 0.001 for Fungi) and between trees (FS17 + Kalamata; R = 0.223, *p* = 0.01 for Bacteria; R = 0.322, *p* = 0.003 for Fungi), with high and low abundance of *Xylella*, although these latter dissimilarities were less supported, as showed by a low R-value. In contrast, no significant differences were found on both bacterial and fungal community composition between cultivars (FS17 vs. Kalamata). The PERMANOVA analysis corroborated these results, by showing that the variability on bacterial composition was mainly explained by season (26.6%, *p* = 0.001) and abundance of *Xylella* (12.2%, *p* = 0.001), while the cultivar only explained 3.3% of the total bacterial variation, which was not statistically significant (*p* = 0.252). Similarly, the fungal composition in olive tree xylem was mainly explained by season and *Xylella* abundance, being responsible for 26.7% (*p* = 0.001) and 16.0% (*p* = 0.002) of the total variation, respectively. Cultivar explained 5.3% of the fungal variation, but the result was not statistically significant (*p* = 0.050).

In addition, 16S rRNA gene sequencing confirmed the evidence recovered from the WMSS showing that *Xylella* tends to occupy the whole xylem niche negatively affecting the rest of the Bacteria community. Indeed, considering the two time points, the average ratio of *Xylella* over total Bacteria increased from 10.3% to 20.5% for FS17 and from 13.2% to 45% for Kalamata (Table 2). Thus, as observed from WMSS analysis, FS17 was able to better restrain the multiplication of the bacterium than Kalamata.

**Figure 8:**
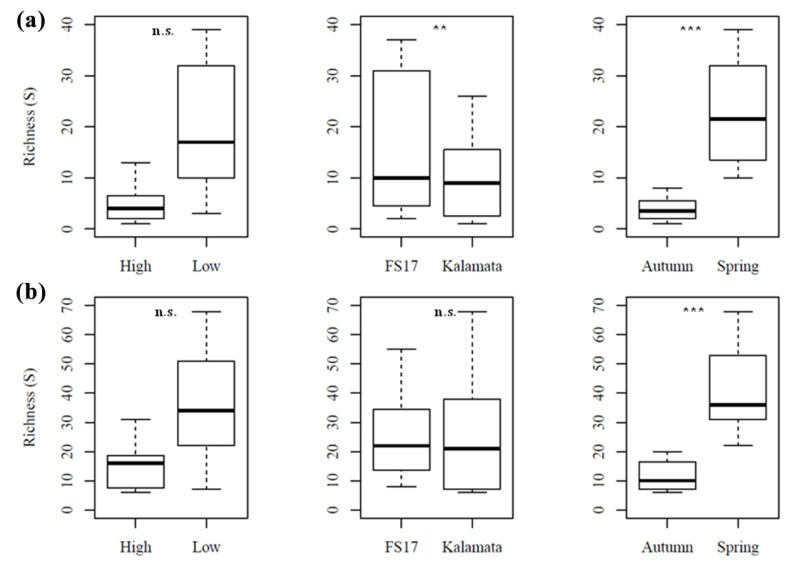
Richness of (**a**) bacterial and (**b**) fungal communities occurring in the xylem of olive trees in relation to *Xylella* abundance (high vs. low), host cultivar (FS17 vs. Kalamata), and season (Spring vs. Autumn). Boxplots depict medians (central horizontal lines), the interquartile ranges (boxes), and 95% confidence intervals (whiskers). Statistical differences between pairs of values are showed (n.s., not significant; ** *p* < 0.01; *** *p* < 0.001).

The bacterial species richness was significantly different between cultivars (LR chi-square = 7.05, *p* < 0.01) and seasons (LR chi-square = 105.57, *p* < 0.001), being higher in cultivar FS17 and in Spring, than in cultivar Kalamata and in Autumn, respectively (Figure 8a). The richness of fungal endophytes only differed significantly (LR chi-square = 120.14, *p* < 0.001) between seasons, being higher in Spring than in Autumn (Figure 8b).

### 2.5. Bacteria/Fungi Genera Shaping the Olive Xylem Microbiome

**Figure 9:**
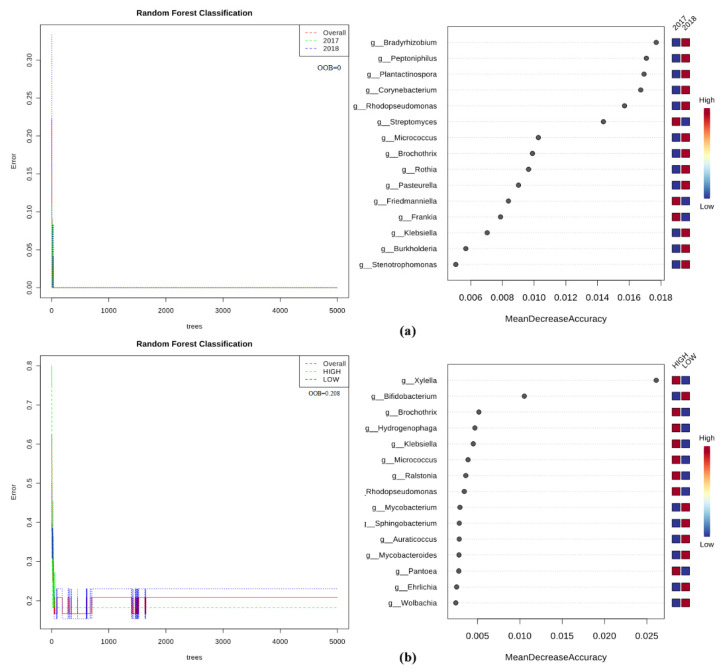
Graphical summary of the random forest analysis of the bacterial community. Significant genera are ranked in decreased order according to their mean decrease accuracy. Color map indicates abundance (red)/scarcity (blue) of genera characterizing samples with high or low *Xylella* abundances according to (**a**) the season or (**b**) *Xylella* experimental factors tested. The out-of-bag (OOB) values are reported, and the analysis was trained with 5000 trees.

To identify factors shaping the bacterial microbiomes, a random forest (RF) analysis, which allows ranking the importance of bacterial genera, was carried out using data from WMSS. The RF graphical output shows that Spring 2017 and Autumn 2018 microbiomes were strongly characterized by 12 microbial features (genera). Indeed, *Bradyrhizobium*, *Peptoniphilus*, *Plantactinospora*, *Corynebacterium*, and *Rhodopseudomonas* genera characterize the Autumn microbiomes, while *Streptomyces*, *Friedmanniella*, and *Frankia* those of Spring (Figure 9a). Besides the obvious *Xylella*, all other identified genera *Brochotrix*, *Hydrogenophaga*, *Klebsiella*, *Micrococcus*, *Ralstonia*, and *Pantoea* were significantly but weakly associated with olives with high *Xylella* abundance. Conversely, only *Bifidobacterium* (Figure 9b) moderately associates with olives having a low *Xylella* abundance. Whereas not significant genera were identified by the comparison of the two cultivars (not shown). Similar results were obtained excluding *Xylella* from the dataset.

**Figure 10:**
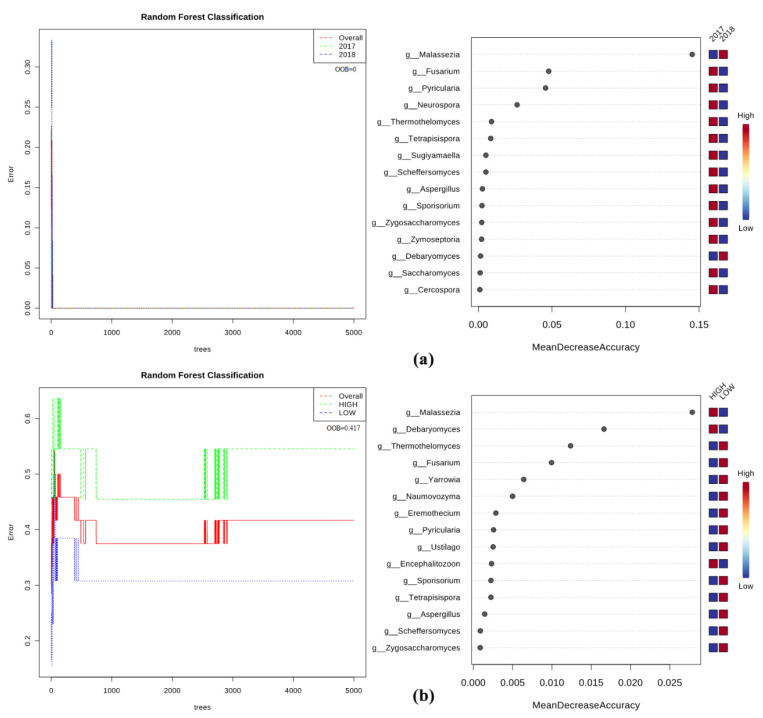
Graphical summary of the random forest analysis of the fungal community. Significant genera are ranked in decreased order according to their mean decrease accuracy. Color map indicates abundance (red)/scarcity (blue) of genera characterizing samples with high or low *Xylella* abundances according to (**a**) the year or (**b**) *Xylella* experimental factors tested. The OOB values are reported, and the analysis was trained with 5000 trees.

**Figure 11:**
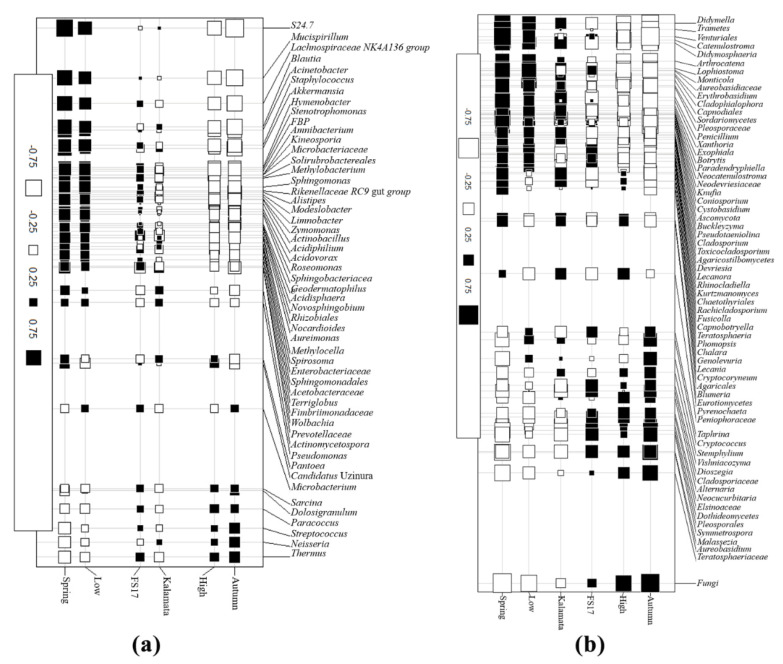
Co-inertia factorial map showing positive (■) and negative (□) relationships between (**a**) bacterial and (**b**) fungal genera from olives with different *Xylella* abundance (high vs. low), from different cultivar (FS17 vs. Kalamata), and diverse seasons (Spring vs. Autumn). Square size is proportional to correlation intensity. The Fungi co-inertia factorial map shows only the genera with correlations higher than 0.25 (both positive and negative).

Random forest analysis perfectly confirmed the main role of the season in differentiating the olive fungal microbiomes as the performance of the test was very significant (i.e., the grown trees early overlap and the out-of-bag (OOB) value is 0) (Figure 10a). The genus *Malassezia* was significantly associated with the Autumn 2018 microbiomes while the genera *Fusarium* and *Pyricularia* were found among the Spring 2017-associated microbiomes. Moreover, *Xylella* role in shaping the Fungi microbiome was present although limited, as shown by the low performance (OOB = 0.417) of RF analysis (Figure 10b). However, also testing *Xylella* abundance as an experimental factor, *Malassezia* was identified as an associated genus, in addition to *Debaromyces*. Conversely, fungal genera associated to microbiomes having a low *Xylella* abundance belonged to *Thermothelomyces*, *Fusarium*, *Yarrowia*, and *Naumovozyma* (Figure 10b). Similarly, to identify a set of bacterial/fungal genera associated to *Xylella* (high vs. low), host cultivar (FS17 vs. Kalamata), and season (Spring vs. Autumn), a co-inertia analysis was performed either for bacteria (Figure 11a) or fungi (Figure 11b) using data from 16S and ITS1 rRNA gene sequencing, respectively. The results showed a set of bacterial genera positively associated with a high abundance of *Xylella*, with *Thermus*, *Paracoccus*, *Sarcina*, *Neisseria*, and *Streptococcus* being the predominant genera. Members of these genera were also found to be positively correlated with Autumn 2018. In contrast, olive tree samples from Spring 2017 and with low abundance of *Xylella*, were found to be positively correlated with the presence of members belonging mostly to *Mucispirillum*, *Lachnospiraceae*, *Blautia*, *Staphylococcus*, and one unknown bacteria (S24-7). Olive cultivars could not be differentiated based on the association of specific bacterial endophytes. Co-inertia analysis also revealed that a set of fungal genera were positively correlated to each season or *Xylella* abundance (high/low), whereas host plant cultivars were not differentiated by fungal endophytes (Figure 11b). In particular, the fungal genera *Peniophoraceae*, *Malassezia*, *Alternaria*, *Neocucurbitaria*, and *Elsinoaceae* were found to be the most positively correlated to trees with a high abundance of *Xylella* and collected in Autumn 2018. In contrast, the fungal genera *Catenulostroma*, *Monticola*, *Arthrocatena*, and *Didymella* were the most positively correlated to trees with a low abundance of *Xylella* and with Spring 2017.

## 3. Discussion

An in silico analysis of the xylem microbiome of field-grown olives exposed to natural *X. fastidiosa* infection was performed with two sequencing approaches, a classical 16S/ITS rRNA gene amplicon sequencing and WMSS. Both approaches have their pros and cons, which mainly rely on the analysis of a single gene, consolidated pipelines for the analysis, and low costs for 16S/ITS rRNA gene, opposed to higher sequencing depths, costs, and data recovery using WMSS [47,48,49,50].

To the best of our knowledge, this is the first study investigating the WMSS analysis of the xylem microbiomes of trees infected with *X. fastidiosa*, using non-targeted sequencing. Although the recovered sequence data largely originated (up to 99.92%) from the olive genome, the depth of WMSS was exhaustive of the bacterial, fungal, and archaeal endophytic microbiome of these plants, as shown by the rarefaction analysis and the taxa identified, which are similar to those reported in other studies in olive (see below). WMSS returned reads classified in the Bacteria, Archaea, and Eukarya kingdoms, this latter composed of plant and fungal taxa. Virus-associated reads were found, but their study was abandoned not only for the paucity of the viral-sequences recovered, but also considering that the majority (~65%) of the plant-associated viruses are RNA-viruses, and localize in the phloem or the parenchyma of the infected hosts, while our analysis targeted the xylem tissues. Indeed, the presence of viruses in the xylem is a poorly investigated subject of research and evidence reports the release in the extracellular space of “virus-replication factories” of RNA-genome species which, however, seems to contribute to the systemic Virus spread, while the presence of intact viral particles is not completely demonstrated [51,52].

Bacteria were found to be the main class of microorganisms inhabiting the olives endophyte microbiome, reaching 99.14% of all microbe-associated reads, a finding previously reported in WMSS analyses of *Arabidopsis thaliana* microbiome [27,53] and likely explained by the larger size of fungal genomes that, together with the lack of sufficient fungal genomic data available in databases, limit their classification. Conversely, 16S/ITS rRNA gene analysis returned the opposite picture, showing that the majority of classified reads were from fungi, a result likely biased by the high percentage (up to 99%) of reads amplified by the 16SrRNA gene primers that indeed belonged to the olive genome. The same problem has been reported by many other researchers being most critical in plant above-ground green tissues, including in olive trees [39]. Despite the differences in bacteria/fungi relative composition, both sequencing approaches (WMSS and 16S rRNA gene) identified *Proteobacteria*, *Actinobacteria*, *Firmicutes*, and *Bacteriodetes* as the most dominant phyla, in agreement with previous studies [39,40]. A good correlation in the classification of the bacterial taxa was obtained between the two sequencing approaches at higher taxonomical levels (phylum, order, and class), while it decreased when lower levels were considered, likely due to the different depth of the data. These four dominant phyla were indeed found to be predominant in the microbiomes recovered from *Xylella*-infected Leccino and Cellina di Nardò trees [46], as well as in the endophytic microbiome of healthy olive trees [41]. The dominant fungal phyla were *Ascomycota* and *Basidiomycota*, confirming previous ITS microbiome analyses [42,44,46,54], but the agreement between WMSS and ITS rRNA gene approaches was not maintained as different taxa abundances were classified at all taxonomic levels. The occurrence of Archaea was confirmed by WMSS analysis and, as in the study of Müller et al. [39], *Euryarchaeota*, *Crenarchaeota*, and *Thaumarchaeota* were the most represented phyla. Little is known about the role of these microbes in plant microbiomes, in which they have been found as main constituents [55]. Perhaps, the xylem microbiome is an appropriate ecological niche for these extremophiles and notably, in our analysis, it is particularly rich in several methanogenic genera that thrive in these conditions.

When the whole microbial communities are considered, in contrast with previous studies [56,57], no cultivar effect on bacterial and fungal endophytic assemblages was found using both sequencing approaches. However, our result is in agreement with the results of a recent microbiome investigation on the *Verticillium*-olive pathosystem showing similar root endosphere and rhizosphere microbial communities between susceptible and tolerant cultivars [37]. In our study, the negligible host cultivar effect on microbiome composition might be explained by the high presence/abundance of *Xylella* in the orchards surveyed that seems to have overshadowed the effect of host cultivar in shaping endophytic microbial communities. Indeed, the level of *Xylella* abundance showed to have a strong effect on endophytic assemblage, explaining 12.2% or 16.0% of the variance in bacterial and fungal diversity across samples, respectively in the 16S/ITS1 rRNA gene analysis, while in WMSS analysis *Xylella* represented 72.14% of the bacterial endophytic microbiome in the orchard. Our analysis shows that *Xylella* abundance largely increased over time, tending to occupy the whole bacterial niche of the xylem. However, a major and significant effect of *Xylella* is exerted on the bacterial community of the cultivar Kalamata, thus showing that FS17, although infected, is more resilient to the presence of *X. fastidiosa*. Thus, we hypothesized that olive tree-associated microbial assemblages are probably shaped by niche-based processes, being the interaction between *Xylella* and the native microbiome a key driver of these selective forces, as previously suggested by McNally and Brown [58]. In both olive cultivars, an increase in *Xylella* abundance over time was observed, which seems to have a large impact on the rest of the microbial community, except for Archaea. It is possible that during the colonization of plant tissues, *Xylella* utilizes methods to displace resident species from their established niches to create its own niche. Such microbes that are likely to exert a high influence on the structure of microbial communities have recently been termed as keystone species [59].

Season was the most important parameter for shaping the bacterial and fungal communities in both WMSS and 16S/ITS1 rRNA gene analyses. Likewise, seasonal variations were found to affect bacterial [60] and fungal [61] endophytic communities of other plant species, including olive trees [44]. The highest abundance and richness of Bacteria and Fungi observed during Spring may be due to climatic conditions that favor the growth or dispersal of microorganisms, as previously suggested [44]. However, the decrease of microbial diversity in Autumn might also be a response to the increase in *Xylella* abundance on the endophytic community of olive trees from Spring 2017 to Autumn 2018. Within these communities, *Xylella* may compete with their neighbors for space and resources, which may lead to changes in microbial diversity [62]. Archaea communities, only detected in WMSS, did not significantly change according to the season, *X. fastidiosa* infection status, and cultivars, perhaps because of their higher ability to adapt to changing environmental conditions. Any conclusion about their role and microbial interactions in the olive microbiome is very speculative.

Unfortunately, we did not find a consensus between both sequencing strategies concerning the identification of bacterial and fungal consortia strongly associated with *Xylella* abundance, the olive cultivar, or the season. With 16S/ITS1 rRNA gene a set of bacterial (*Thermus, Paracoccus*, *Sarcina*, *Neisseria*, and *Streptococcus*) and fungal (*Peniophoraceae*, *Malassezia*, *Alternaria*, *Neocucurbitaria*, and *Elsinoaceae*) taxa, at genus/family level, highly positively correlated with the high abundance of *Xylella,* was found. The genus *Thermus* can be found in many diverse habitats [63] including insects’ gut microbiome [64], and plants’ microbiome [65] with no specifically recognized function. *Paracoccus* genus includes species with plant growth-promoting traits [66]. *Sarcina* has been identified as part of animals’ gut microbiota [67]. Members of the genera *Neisseria* [68], *Streptococcus* [69], *Malassezia* [70], and *Neocucurbitaria* [71] are mostly described as human pathogens, being not mentioned in literature for their association to plants. The genus *Alternaria* includes both plant-pathogenic and saprophytic species and is one of the most well-known fungal genera that produces diverse secondary metabolites, including toxins [72] and antimicrobial compounds [73]. The family *Peniophoraceae* comprises saprophytic Fungi, whose role in plants is still not known [74]. The *Elsinoaceae* family is not well-studied but it is known to include plant pathogens [75]. This lack of consistency in cultivar- or resistance-associated Bacteria was also revealed in a companion paper [76] where endophytes, and among the others, members of the *Methylobacterium* and *Curtobacterium* genera previously indicated as potential biocontrol endophytes [31,32], were isolated from the same FS17 and Kalamata olives. All the isolated genera (*Methylobacterium*, *Sphingomonas*, *Curtobacterium*, *Novosphingobium*, *Frondihabitans*, *Agrococcus*, and *Micrococcus*) were identified in WMSS and the majority of them in 16S rRNA gene analysis, but none were found having in vitro antagonistic activity against *Xylella* [76]. In addition, the present work does not identify the association of microbial consortia with the host resistance, leaving open the possibility that other plant traits are responsible for controlling *Xylella* population size and its pathogenic effects.

Among the season-associated Bacteria, WMSS identified the nitrogen-fixing *Bradyrhizobium* genus [77], already reported in olive by [41], the anaerobes *Peptoniphilus* [78], which include human pathogenic species, and the *Streptomyces* genus, whose members are known as bioremediators and plant-growth-promoters [79]. The only WMSS and 16S/ITS rRNA gene shared genus was *Malassezia* that was positively associated to plants with high *Xylella* abundance.

In conclusion, the bacterial and fungal communities in olive trees xylem appeared to be more tightly structured by season and *Xylella* abundance, than by host cultivar, probably due to the high pressure of inoculum in the orchard where olive trees were sampled. We hypothesized that *Xylella* interacts with the host and the native microbiome dynamically, being responsible for shaping the whole microbial community. However, this effect was variable depending on host cultivar, being microbiome-associated Kalamata was more prone to change than those of cultivar FS17, due to the presence of *Xylella*. Indeed, *Xylella* colonization is significantly more extensive in Kalamata than FS17, which confirms, together with the limitedness of symptoms, the traits of resistance identified in the latter cultivar. Altogether, these results suggest that other mechanisms, likely controlling *Xylella* population size and its pathogenic effects by genetic [13] or anatomic [7] traits, may be responsible for this phenotype.

## 4. Materials and Methods

### 4.1. Collection of Plant Samples

The endophytic microbiome colonizing the xylem tissues was analyzed from 12 field-grown olive trees of the susceptible (Kalamata) and resistant (FS17) cultivars (six each) exposed to natural *X. fastidiosa* infections. Trees were sampled and analyzed twice, in Spring 2017 when infections were still confined with the bacterium in most cases close or under the limit of detection of the qPCR assay and trees being mostly symptomless, and then in Autumn 2018 when the infections reached detectable population levels in trees of both cultivars, with trees of the cultivar Kalamata showing manifest branch desiccations while canopies of the FS17 trees were still symptomless or showing very mild desiccations (i.e., tree FS43). More specifically, samples were collected during April 2017 and November 2018 to take into account the incubation period of the infections [80], and concomitantly to evaluate the change in the microbiomes of resistant and susceptible cultivars with respect to *Xylella* infections.

Samples, consisting of young olive twigs (approximately 0.5 cm diameter), were collected in April 2017 (Spring) and November 2018 (Autumn) from olive trees located in the *X. fastidiosa*-outbreak area in Apulia, in the municipality of Sannicola (40°07′13.77” N, 18°02′40.51” E, Lecce, Italy). Trees from the cultivars FS17 and Kalamata, approximately of the same age (15 years old), were in distinct rows of the same orchard, under the same agricultural management practices. Samples were collected from six trees of each cultivar in 2017 and the same trees in 2018. Following EPPO PM 7/24 (4) standard guidelines [81], 10 twigs of about 0.5 cm in diameter were collected from each tree in the mid part of the canopy, from the four cardinal points, avoiding tissues in an advanced stage of desiccation. Samples were immediately stored in sealed plastic bags and kept refrigerated at 4 °C to avoid dehydration until later processing in the laboratory.

### 4.2. Extraction of Total DNA and Detection of Xylella Fastidiosa

For microbiome DNA extraction from xylem tissue, twigs from each tree sample were cut into 10-cm-long pieces and washed with running tap water, before surface sterilization by sequential dipping in 2% sodium hypochlorite for 2 min, 70% ethanol for 2 min, and three rinses in sterile distilled water. Aliquots of the sterile distilled water used in the final rinse were plated onto tryptic soy agar (TSA). After incubation at 25 °C for 15 days no colonies were apparent, thus confirming the efficacy of the disinfection procedure [82]. After surface disinfection, the end of each twig section and the bark were removed and the debarked tissue was scraped until the hard xylem was exposed, with a sterile scalpel. A total of 1 g of xylem chips was weighed from each tree, placed in a sealed sterile bag (BIOREBA AG, Switzerland) containing 10 mL of hexadecyltrimethylammonium bromide (CTAB), and macerated with a Homex homogenizer (BIOREBA AG, Switzerland). Sample processing was performed in sterile conditions within a flow hood chamber. Samples were further processed for total DNA extraction, performed according to Loconsole et al. [83] and followed by treatment with 50 µg/mL RNase A (Zymo Research Corporation, Orange, CA, USA).

The presence of *X. fastidiosa* in the DNA extracts was assessed by quantitative polymerase chain reaction (qPCR) according to the protocol previously described by Harper et al. [84], using primers XF-F (5′-CACGGCTGGTAACGGAAGA-3′), XF-R (5′-GGGTTGCGTGGTGAAATCAAG-3′), and XF-P probe (5′-FAM-TCGCATCCCGTGGCTCAGTCC-BHQ1-3′). The qPCR reactions were performed on a CFX 96™ Real-Time System (BioRad Laboratories, Hercules, CA, USA), with TaqMan^®^ Fast Advanced Master Mix (Thermo Fisher Scientific, Waltham, MA, USA), using the following cycling conditions: 95 °C for 5 min, then 40 cycles of 94 °C for 10 s and 62 °C for 40 s. Estimated *X. fastidiosa* population size, corresponding to each Cq value, was inferred by a standard calibration curve. The linear regression equation was computed from a triplicate assay using DNA extracted from 10-fold serial dilutions of bacterial suspension, ranging from 10^7^ to 10^2^ CFU/mL, and spiked in homogenized tissues of non-infected olives.

Statistical comparison of the average estimated *X. fastidiosa* population size among the four different samplings (i.e., FS17 April 2017, Kalamata April 2017, FS17 November 2018, Kalamata November 2018) was performed by one-way analysis of variance (ANOVA), followed by Tukey’s post-hoc pairwise comparison. To ensure that the assumptions required for standard parametric analysis of variance were satisfied, the normal distribution of data had been preliminarily ascertained by the Shapiro–Wilk’s test, and homogeneity of variance assessed according to Levene’s test. In all analyses, the null hypothesis was rejected at the 0.05 α-level.

### 4.3. Whole Metagenome Shotgun Sequencing and Bioinformatic Analysis

WMSS was performed with the Illumina 2 × 150 bp format using the TruSeq DNA PCR-free protocol (Illumina Inc., San Diego, CA, USA) that allows a representation of the underlying species composition and relative abundances in a sample without the introduction of PCR bias. Library preparation and sequencing were outsourced to Macrogen Europe (the Netherlands) for tissues sampled in 2017 and to LGC Biosearch Technologies (Germany) for tissues sampled in 2018. The raw reads obtained were quality checked and, whenever required, adaptor sequences were trimmed out using FastQC tool (Andrews, 2010) and reads with a final length <20 bases were discarded. Taxonomic profiling of the raw Illumina read dataset was carried out with Kraken, an ultrafast metagenomic sequence classification tool (Wood and Salzberg, 2014), toward a Kraken database, built using a custom Perl script [85] and the default 31 *k*-mer. The obtained database consisted of 687 sequences from Archaea, 1337 chromosome sequences plus 8078 complete genome sequences from Bacteria, 249 sequences from Fungi, and 7540 complete genome sequences of Viruses. This initial analysis classified the majority of reads as belonging to the Fungi kingdom, but an in-depth BLASTn search of this fraction disclosed that these reads indeed corresponded to plant DNA sequences. We, therefore, discarded these data and successively re-classified reads with Kraken 2, the newest version of the software [86], using a custom-made Kraken database that includes: 533 (Archaea), 38,758 (Bacteria), 11,953 (Viruses), 1472 (Fungi), and 621,633 (plant) sequences, respectively, and a longer 41 *k*-mer. Raw reads from each sample were searched against this custom-made Kraken database, resulting in their classification at different taxonomic levels.

Plant reads corresponding to ribosomal RNAs were manually eliminated from the Kraken files after being identified by BLASTn analysis. Kraken 2.mpa files were imported in MEGAN [87] from which separate Bacteria, Fungi, Archaea, and Viruses comparison.txt files were produced by using absolute read counts and ignoring all unassigned reads. To correct for the different sequencing depth of libraries, microbial reads (i.e., Bacteria, Fungi, Archaea, and Viruses) from the respective comparison.txt files were normalized according to Regalado et al. [27], by using plant reads as internal spike-in. Briefly, Kraken 2-classified data were normalized according to the formula

$$Xnorm_{i}=\hat{P}\cdot \frac{Xraw_{i}}{P_{i}}$$

were *Xnorm_i_*, *P*, *Xraw_i_*, and *P_i_* stand respectively for the normalized reads in sample_i_, the average number of plant reads among all samples, the raw number of reads assigned to a microbial taxon, and the number of plant reads in that sample. Microbe-normalized *comparison.txt* files containing data from all libraries were imported in MicrobiomeAnalyst [88,89] where taxa having less than a 20% prevalence and with a minimum of 10, 50, 10, and 10 reads for Archea, Bacteria, Fungi, and Viruses, respectively, were filtered out (a 20% prevalence filter with a minimum of 50 reads means that at least 20% of its values should contain at least 50 reads in the case of Bacteria). Data having a low variance (i.e., taxa constant throughout the samples) were filtered by applying a 10% inter-quantile range measure of variance and were further normalized according to clr transformation, to take into account the compositional nature of the metagenomic data [90].

Good’s coverage index (which estimates the probability that the next read will belong to an existing taxon) and biomarkers characterizing *Xylella* abundance (high vs. low), cultivar (FS17 vs. Kalamata), and season (Spring vs. Autumn), were estimated by random forest analysis, by using MicrobiomeAnalyst with default parameters of 5000 trees to grow and randomness setting parameters. Clr-transformed data were ordinated by principal component analysis (PCA) using a variance-covariance matrix and the significance of the clustering was tested by analysis of group similarities (ANOSIM), computed using the Euclidean index of distance similarity, by the PAST3 software [91].

Pearson’s correlation analysis was performed to analyze the correlation between the proportion of *Xylella* to total bacterial reads and the population size, as estimated by qPCR. The assumption of normal distribution was preliminarily assessed by the Shapiro–Wilk’s test. Statistical significance was accepted at the α = 0.05 level.

### 4.4. 16S and ITS1 rRNA Gene Library Sequencing and Bioinformatic Analysis

The DNA was analyzed by high throughput sequencing using Illumina MiSeq platform with the paired-end option (2 × 250 bp). Bacterial community present in xylem wood shavings were assessed by sequencing the V4 region of the 16S gene of rRNA gene with the primer pairs 515f/806rB [92], using services available at the Instituto Gulbenkian de Ciência (IGC, Portugal). For the fungal community, the ITS1 region of rRNA gene was amplified with the primer pairs ITS1F/ITS2 [92] and custom sequenced at LGC Biosearch Technologies (Germany) facilities. The raw sequencing data were first subjected to a quality report visualized in FastQC. Based on the quality scores, read trimming was performed in Sickle [93] to eliminate the incorrectly placed bases in the 3′-end and 5′-end regions, to obtain a greater read quality. Singles, i.e., unpaired reads, for which only the reverse or forward sequence was approved on the quality report, were also eliminated, keeping only good quality paired reads for the following analysis. After trimming, read errors constructed during the sequencing process were corrected using SPAdes [94]. The merge of overlapping paired-end reads was performed using USEARCH [95]. A new quality report was then performed with FastQC. From this report, read filtering parameters based on expected amplicon size were determined. The filtering was applied using ea-utils [96]. Clustering of reads in OTUs, and their taxonomic assignment at 97% similarity, was performed with MICCA [97]. Taxonomic classification was assigned by using the reference database SILVA version 132 [98,99] for the Bacteria and UNITE version 8.0 [100,101] for the Fungi. Unassigned OTUs and those that were identified as mitochondrial or plastid DNA, as well as OTUs with low abundance (i.e., less than five or 10 reads for Bacteria and Fungi, respectively), were removed from further analyses. All statistical analyses were performed by using this dataset, where the *Xylella* species data were excluded.

The effect of the abundance of *Xylella*, host cultivar, and season in the microbiome diversity was determined by evaluating the richness by using the vegan package [102] and *diversity* function in R software [103]. To compare the differences between means, one-way ANOVA, followed by Tukey’s post-hoc test (significance level α = 0.05) was performed by using the same software.

Non-metric multidimensional scaling (NMDS) was performed using Bray–Curtis index with normalized OTU matrix, to calculate the average dissimilarity in the composition of bacterial or fungal communities in olive tree xylem due to different *Xylella* abundance (high vs. low), host cultivar (FS17 vs. Kalamata), and season (Spring vs. Autumn). Kruskal’s stress was used to estimate the model’s goodness of fit, with a commonly accepted value when lower than 0.2 [104]. ANOSIM analysis of similarity was also performed, using Bray–Curtis distance matrices, to find significant differences between the bacterial or fungal community groups observed in NMDS ordination. This analysis generates a *p*-value (significant if ≤ 0.05) associated to an R-value, which ranges from 0 (completely similar) to 1 (completely different) [105]. Both NMDS and ANOSIM analyses were performed using the vegan package (*metaMDS* and *anosim* functions, respectively) in R software.

Contribution of *Xylella* abundance, host cultivar, and season to the xylem microbiome community structure was deciphered by using permutational multivariate analysis of variance (PERMANOVA), which was performed using the function *adonis* in the R vegan package. Additionally, a co-inertia analysis (CIA) was conducted to determine the relationship between bacterial/fungal genera and the abundance of *X. fastidiosa*, host cultivar, or season. This analysis was performed in R, using the *ade4* package [106] and the *table.value* function to visualize the results.

Raw sequence reads and related metadata were deposited at the Sequence Read Archive (National Center for Biotechnology Information, USA National Library of Medicine, Bioproject #PRJNA629675: https://www.ncbi.nlm.nih.gov/bioproject/PRJNA629675).

## Figures and Tables

**Table 1:** Summary of the Illumina sequencing and reads classification in the two phenological stages (spring: April 2017, autumn: November 2018). * Numbers are from Kraken 2 classification. (**) Numbers are from MEGAN classification and successive (***) normalization to *Plants* reads. Percentages of bacteria, fungi, archaea, and viruses are related to the total reads microbes.

Season	Cultivar	Sample Name *	Raw Total Reads *	Reads Classified * (%)	Reads Unclassified * (%)	Plants Reads ** (%)	Total Reads Microbes **	Bacteria *** (%)	Fungi *** (%)	Archaea *** (%)	Viruses *** (%)	*Xylella* Cq	*Xylella* CFU/ml	% *Xylella*/Bacteria Reads	% Average *Xylella*/ Bacteria
Spring April 2017	FS17	FS1-1	50,852,732	49,963,303 (98.2)	889,429 (1.75)	49,808,696 (99.69)	259,160	249,675 (96.34)	6893 (2.66)	1735 (0.67)	857 (0.33)	28.30	34,100	0.37	2.32
		FS1-3	46,489,102	45,588,956 (98.0)	900,146 (1.94)	45,428,720 (99.65)	261,865	251,689 (96.11)	7435 (2.84)	1932 (0.74)	809 (0.31)	31.20	4430	0.06	
		FS1-10	52,171,471	51,356,470 (98.4)	815,001 (1.56)	51,202,008 (99.70)	268,515	261,563 (97.41)	4474 (1.67)	1623 (0.60)	855 (0.32)	27.00	84,900	0.40	
		FS1-18	54,871,547	53,962,289 (98.3)	909,258 (1.66)	53,845,628 (99.78)	205,545	198,322 (96.49)	4550 (2.21)	1801 (0.88)	872 (0.42)	30.10	9610	0.18	
		FS1-43	42,746,629	42,110,247 (98.5)	636,382 (1.49)	42,020,764 (99.79)	158,999	154,117 (96.93)	2764 (1.74)	1277 (0.80)	841 (0.53)	22.90	1,520,000	12.87	
		FS1-45	39,194,825	38,375,168 (97.9)	819,657 (2.09)	38,227,260 (99.61)	240,666	230,319 (95.70)	7825 (3.25	1548 (0.64)	974 (0.40)	35.10	286	0.02	
	Kalamata	Kal1-53	38,656,227	38,101,308 (98.5)	554,919 (1.44)	38,028,096 (99.81)	130,362	126,247 (96.84)	2375 (1.82)	980 (0.75)	760 (0.58)	36.10	142	0.05	8.69
		Kal1-54	44,921,146	43,839,640 (97.5)	1,081,506 (2.41)	43,586,856 (99.42)	405,349	391,036 (96.47)	11,283 (2.78)	2135 (0.53)	895 (0.22)	28.50	29,600	0.10	
		Kal1-55	39,692,637	39,116,264 (98.5)	576,373 (1.45)	39,009,116 (99.73)	178,457	172,754 (96.80)	2438 (1.37)	1077 (0.60)	2188 (1.23)	33.10	1170	0.02	
		Kal1-57	42,445,268	41,813,669 (98.5)	631,599 (1.49)	41,711,976 (99.76)	181,331	176,926 (97.57)	2647 (1.46)	1203 (0.66)	555 (0.31)	31.10	4760	0.22	
		Kal1-65	45,136,293	44,493,779 (98.5)	642,514 (1.42)	44,384,016 (99.75)	194,201	186,304 (95.93)	2783 (1.43)	4058 (2.09)	1056 (0.54)	34.10	577	0.04	
		Kal1-89	44,631,934	43,883,925 (98.3)	748,009 (1.68)	43,692,880 (99.56)	343,522	337,244 (98.17)	3490 (1.02)	1417 (0.41)	1371 (0.40)	20.40	8,790,000	51.73	
Autumn November 2018	FS17	FS2-1	33,462,404	32,788,919 (97.9)	673,485 (2.01)	32,749,304 (99.88)	65,201	59,793 (91.71)	3284 (5.04)	989 (1.52)	1135 (1.74)	35.10	286	0.95	31.48
		FS2-3	32,092,733	31,396,019 (97.8)	696,714 (2.17)	31,371,784 (99.92)	38,374	34,366 (89.56)	2222 (5.79)	770 (2.01)	1016 (2.65)	29.96	10,600	4.89	
		FS2-10	33,465,333	32,683,674 (97.6)	781,659 (2.34)	32,631,210 (99.84)	90,038	84,585 (93.94)	3636 (4.04)	934 (1.04)	883 (0.98)	25.50	244,000	46.82	
		FS2-18	32,789,969	32,080,877 (97.8)	709,092 (2.16)	32,029,304 (99.84)	86,409	79,573 (92.09)	4305 (4.98)	957 (1.11)	1574 (1.82)	31.10	4760	15.42	
		FS2-43	42,453,647	41,659,140 (98.1)	794,507 (1.87)	41,142,644 (98.76)	938,782	923,823 (98.41)	10,798 (1.15)	1082 (0.12)	3079 (0.33)	24.57	469,000	83.11	
		FS2-45	31,955,583	31,276,525 (97.8)	679,058 (2.13)	31,230,648 (99.85)	77,562	72,841 (93.91)	2862 (3.69)	857 (1.10)	1002 (1.29)	26.60	112,000	37.68	
	Kalamata	Kal2-53	48,745,550	47,873,736 (98.2)	871,814 (1.79)	47,214,900 (98.62)	1,198,439	1,188,286 (99.15)	5768 (0.48)	1299 (0.11)	3086 (0.26)	24.20	608,000	90.05	52.67
		Kal2-54	33,545,651	32,875,120 (98.0)	670,531 (2.00)	32,841,592 (99.90)	56,343	51,642 (91.66)	2265 (4.02)	738 (1.31)	1698 (3.01)	21.50	4,060,000	34.41	
		Kal2-55	38,914,006	38,071,869 (97.8)	842,137 (2.16)	37,429,772 (98.31)	1,162,983	1,152,955 (99.14)	6234 (0.54)	1318 (0.11)	2476 (0.21)	22.10	2,660,000	88.65	
		Kal2-57	30,275,514	29,663,797 (97.9)	611,717 (2.02)	29,635,620 (99.91)	45,484	40,442 (88.91)	2912 (6.40)	902 (1.98)	1228 (2.70)	21.80	3,290,000	0.45	
		Kal2-65	29,620,002	29,066,308 (98.1)	553,694 (1.87)	29,033,788 (99.89)	52,570	47,964 (91.24)	2148 (4.09)	771 (1.47)	1687 (3.21)	22.20	2,480,000	19.94	
		Kal2-89	25,255,482	24,756,720 (98.0)	498,762 (1.97)	24,597,096 (99.36)	287,446	281,221 (97.83)	2430 (0.85)	729 (0.25)	3066 (1.07)	22.40	2,150,000	82.53	

**Table 2:** Summary of the Illumina 16S and ITS1 rRNA gene sequencing and reads classification in the two phenological stages.

Season	Cv.	Sample Name	Total Raw Reads		Sequences Classified (%)		Sequences Unclassified (%)		Plant Sequences (%)	Bacteria (%)	Fungi (%)	% *Xylella*/ Bacteria	% Average *Xylella*/Bacteria
			16S	ITS	16S	ITS	16S	ITS	16S				
Spring April 2017	FS17	FS1-1	13,604	110,867	13,137 (96.6)	88,348 (79.7)	467 (3.4)	22,519 (20.3)	12,753 (97.1)	384 (2.9)	88,338 (100)	0.8	10.3
		FS1-3	14,415	25,801	13,859 (96.1)	17,759 (68.8)	556 (3.9)	8042 (31.2)	13,386 (96.6)	473 (3.4)	17,759 (100)	0.4	
		FS1-10	14,930	77,958	14,440 (96.7)	59,012 (75.7)	490 (3.3)	18,946 (24.3)	14,253 (98.7)	187 (1.3)	59,012 (100)	1.6	
		FS1-18	12,303	62,221	11,899 (96.7)	31,172 (50.1)	404 (3.3)	31,049 (49.9)	11,826 (99.4)	73 (0.6)	31,172 (100)	2.7	
		FS1-43	10,505	64,245	10,259 (97.7)	34,647 (53.9)	246 (2.3)	29,598 (46.1)	10,125 (98.7)	134 (1.3)	34,647 (100)	56.0	
		FS1-45	12,735	90,246	12,250 (96.2)	63,942 (70.9)	485 (3.8)	26,304 (29.1)	11,772 (96.1)	478 (3.9)	63,942 (100)	0.0	
	Kalamata	Kal1-53	11,780	22,772	11,335 (96.2)	15,618 (68.6)	445 (3.8)	7154 (31.4)	11,296 (99.7)	39 (0.3)	15,618 (100)	0.0	13.2
		Kal1-54	15,279	26,9041	14,598 (95.5)	174,764 (65)	681 (4.5)	94,277 (35.0)	13,807 (94.6)	791 (5.4)	174,761 (100)	0.0	
		Kal1-55	12,827	26,740	12,407 (96.7)	18,577 (69.5)	420 (3.3)	8163 (30.5)	12,156 (98.0)	251 (2.0)	18,577 (100)	0.0	
		Kal1-57	9529	18,716	9259 (97.2)	12,313 (65.8)	270 (2.8)	6403 (34.2)	9222 (99.6)	37 (0.4)	12,313 (100)	0.0	
		Kal1-65	10,275	111,806	9936 (96.7)	67,804 (60.6)	339 (3.3)	44,002 (39.4)	9897 (99.6)	39 (0.4)	67,804 (100)	0.0	
		Kal1-89	12,140	17,612	11,612 (95.7)	15,966 (90.7)	528 (4.3)	16,746 (95.1)	10,808 (93.1)	804 (6.9)	15,966 (100)	79.2	
Autumn November 2018	FS17	FS2-1	151,666	88,600	151,475 (99.9)	78,642 (88.8)	191 (0.1)	9958 (11.2)	15,1200 (99.8)	275 (0.2)	78,642 (100)	0.0	20.5
		FS2-3	246,844	209,701	246,317 (99.8)	188,116 (89.7)	527 (0.2)	21,848 (10.4)	246,201 (99.9)	116 (0.05)	187,853 (99.9)	6.9	
		FS2-10	195,548	219,077	195,319 (99.9)	203,699 (93.0)	229 (0.1)	15,975 (7.3)	195,168 (99.9)	150 (0.1)	203,102 (99.7)	46.0	
		FS2-18	175,303	91,227	175,011 (99.8)	73,264 (80.3)	292 (0.2)	18,023 (19.8)	174,853 (99.9)	158 (0.1)	73,204 (99.9)	12.7	
		FS2-43	92,265	103,006	92,257 (100)	97,801 (94.9)	8 (0)	5205 (5.1)	92,062 (99.8)	195 (0.2)	97,801 (100)	44.1	
		FS2-45	131,843	65,534	131,659 (99.9)	23,987 (36.6)	184 (0.1)	41,709 (63.6)	131,416 (99.8)	243 (0.2)	23,825 (99.3)	13.2	
	Kalamata	Kal2-53	168,034	54,316	167,912 (99.9)	23,089 (42.5)	122 (0.1)	31,227 (57.5)	167,418 (99.7)	494 (0.3)	23,089 (100)	77.9	45.0
		Kal2-54	243,771	69,589	243,266 (99.8)	58,880 (84.6)	505 (0.2)	10,728 (15.4)	243,189 (99.9)	77 (0.03)	58,861 (100)	39.0	
		Kal2-55	121,959	199,625	121,878 (99.9)	143,056 (71.7)	81 (0.1)	56,569 (28.3)	121,179 (99.4)	699 (0.6)	143,056 (100)	81.4	
		Kal2-57	162,670	47,373	162,263 (99.7)	47,368 (100)	407 (0.3)	244 (0.5)	162,224 (99.9)	39 (0.02)	47,129 (99.5)	0.0	
		Kal2-65	69,232	78,285	69,182 (99.9)	39,009 (49.8)	50 (0.1)	39,276 (50.2)	69,143 (99.9)	39 (0.1)	39,009 (100)	15.4	
		Kal2-89	155,471	84,249	155,407 (100)	81,875 (97.2)	64 (0)	2374 (2.8)	155,219 (99.9)	188 (0.1)	81,875 (100)	56.4	

## Supplementary Materials

The following are available online at https://www.mdpi.com/2076-0817/9/9/723/s1, Figure S1: Pearson’s correlation analysis between *X. fastidiosa* average population size and *Xylella*/Bacteria relative read abundance, Figure S2: Average estimated *X. fastidiosa* population size among the four different samplings, Figure S3: Linear correlation between *Xylella* and total Bacteria, Table S1: Good’s coverage index estimation for Bacteria, Fungi and Archaea, Table S2: Results of one-way ANOVA comparing the average estimated *X. fastidiosa* population size among the four different samplings, Table S3: Summary of the ANOSIM statistics comparing Bacteria microbiomes, Table S4: Summary of the ANOSIM statistics comparing Fungi microbiomes, Table S5: Summary of the ANOSIM statistics comparing Archaea microbiomes, File S1: Bacteria abundance, File S2: Fungi abundance, File S3: Archaea abundance, File S4: Virus abundance.
