# Supplementary material for: Differences in the Endophytic Microbiome of Olive Cultivars Infected by Xylella fastidiosa across Seasons
Source: Pathogens. 2020 Sep 2;9(9):723. doi: 10.3390/pathogens9090723 (PMC7558191; doi:10.3390/pathogens9090723)
Supplement: Supplementary file 1 [file pathogens-09-00723-s001.zip › Table S1.docx]

**Table S1**. Good's coverage index estimation for Bacteria, Fungi and Archaea*.*

|  | **Good’s Index** | | |
| --- | --- | --- | --- |
| **Sample** | ***Bacteria*** | ***Fungi*** | ***Archaea*** |
| FS1-43 | 99.67 | 100.0 | 98.45 |
| Kal1-53 | 99.35 | 100.0 | 98.29 |
| Kal1-57 | 99.54 | 100.0 | 98.19 |
| FS1-45 | 99.92 | 100.0 | 99.33 |
| Kal1-65 | 99.5 | 100.0 | 99.55 |
| Kal1-54 | 99.97 | 100.0 | 99.62 |
| FS1-18 | 99.68 | 100.0 | 99.03 |
| Kal1-55 | 99.75 | 100.0 | 97.97 |
| FS1-10 | 99.87 | 100.0 | 98.33 |
| FS1-3 | 99.93 | 100.0 | 99.26 |
| FS1-1 | 99.91 | 100.0 | 99.11 |
| Kal1-89 | 99.92 | 100.0 | 98.54 |
| Kal2-55 | 99.99 | 100.0 | 98.01 |
| Kal2-53 | 99.99 | 100.0 | 98.88 |
| Kal2-54 | 99.59 | 100.0 | 97.54 |
| FS2-43 | 99.98 | 100.0 | 98.73 |
| FS2-45 | 99.73 | 100.0 | 97.13 |
| FS2-1 | 99.57 | 100.0 | 96.91 |
| Kal2-57 | 99.46 | 100.0 | 98.49 |
| Kal2-89 | 99.93 | 100.0 | 97.21 |
| Kal2-65 | 99.59 | 100.0 | 97.52 |
| FS2-18 | 99.75 | 100.0 | 98.14 |
| FS2-3 | 99.45 | 100.0 | 98.08 |
| FS2-10 | 99.76 | 100.0 | 97.86 |
| **AVERAGE** | **99.74** | **100.0** | **98.34** |
