# Supplementary material for: Differences in the Endophytic Microbiome of Olive Cultivars Infected by Xylella fastidiosa across Seasons
Source: Pathogens. 2020 Sep 2;9(9):723. doi: 10.3390/pathogens9090723 (PMC7558191; doi:10.3390/pathogens9090723)
Supplement: Supplementary file 1 [file pathogens-09-00723-s001.zip › Table S2.docx]

**Table S2.** Results of one-way ANOVA comparing the average estimated *X. fastidiosa* population size (Log CFU/ml) among the four different samplings (i.e. FS17 Apr 2017, Kalamata Apr 2017, FS17 Nov 2018, Kalamata Nov 2018). Post-hoc pairwise comparisons were performed using the Tukey’s HSD test. *df*: degrees of freedom. *The mean difference is significant at α = 0.05 level.

| **One-way ANOVA** | | | | | | | |
| --- | --- | --- | --- | --- | --- | --- | --- |
|  | **Sum of Squares** | | ***df*** | | **Mean Square** | **F-value** | **p-value** |
| Between Groups | 22.228 | | 3 | | 7.409 | 4.865 | 0.011* |
| Within Groups | 30.461 | | 20 | | 1.523 |  |  |
| Total | 52.690 | | 23 | |  |  |  |
| **Tukey’s HSD post-hoc pairwise comparisons** | | | | | | | |
| **Pairwise Comparison** | | **Mean Difference** | | **Std. Error** | **p-value** | **95% Confidence Interval** | |
|  |  |  |  |  |  | **Lower Bound** | **Upper Bound** |
| FS17 Apr 2017 | FS17 Nov 2018 | 0.44268 | | 0.71252 | 0.924 | - 1.5516 | 2.4370 |
|  | Kalamata Apr 2017 | - 0.09006 | | 0.71252 | 0.999 | - 2.0844 | 1.9042 |
|  | Kalamata Nov 2018 | - 2.05567 | | 0.71252 | 0.042* | - 4.0500 | - 0.0614 |
| FS17 Nov 2018 | Kalamata Apr 2017 | 0.53274 | | 0.71252 | 0.877 | - 1.4616 | 2.5270 |
|  | Kalamata Nov 2018 | -1.96560 | | 0.71252 | 0.054 | - 3.9599 | 0.0287 |
| Kalamata Apr 2017 | Kalamata Nov 2018 | -2.49835 | | 0.71252 | 0.011* | - 4.4927 | - 0.5040 |
