# Supplementary material for: Differences in the Endophytic Microbiome of Olive Cultivars Infected by Xylella fastidiosa across Seasons
Source: Pathogens. 2020 Sep 2;9(9):723. doi: 10.3390/pathogens9090723 (PMC7558191; doi:10.3390/pathogens9090723)
Supplement: Supplementary file 1 [file pathogens-09-00723-s001.zip › Table S3.docx]

**Table S3**. Summary of the ANOSIM statistics comparing Bacteria microbiomes *per* season, cultivar or *Xylella* abundance. N.S.: not significant (*p > 0.05*). n.d.: not determined; / comparison not possible. Significant (*p*<0.05) values are in bold.

| **ANOSIM** | | **Spring + Autumn** | | **Spring** | | **Autumn** | |
| --- | --- | --- | --- | --- | --- | --- | --- |
|  |  | **R** | ***p*-value** | **R** | ***p*-value** | **R** | ***p*-value** |
| Total FS17+Kalamata | Low vs High | **0.2376** | **0.0055** | 0.08478 | 0.275 | **0.3504** | **0.0155** |
| FS17 |  | 0.2424 | 0.0774 | n.d. | | n.d. | |
| Kalamata |  | **0.4611** | **0.0081** | n.d. | | n.d. | |
| Total High+Low | FS17 vs Kal | 0.00662 | 0.349 | 0.15 | 0.1528 | 0.1093 | 0.1541 |
| High |  | N.S. | | n.d. | | n.d. | |
| Low |  | N.S. | | n.d. | | n.d. | |
| Total FS17+Kalamata | Spring vs Autumn | **0.5165** | **0.0001** | / | / | / | / |
| FS17 |  | **0.5481** | **0.0052** | / | / | / | / |
| Kalamata |  | **0.6111** | **0.002** | / | / | / | / |
