# Supplementary material for: Differences in the Endophytic Microbiome of Olive Cultivars Infected by Xylella fastidiosa across Seasons
Source: Pathogens. 2020 Sep 2;9(9):723. doi: 10.3390/pathogens9090723 (PMC7558191; doi:10.3390/pathogens9090723)
Supplement: Supplementary file 1 [file pathogens-09-00723-s001.zip › Table S4.docx]

**Table S4.** Summary of the ANOSIM statistics comparing Fungi microbiomes of both seasons or separately *per* seasons or cultivars. N.S.: not significant (*p>0.05*). n.d.: not determined; / comparison not possible.

| **ANOSIM** | | **Spring + Autumn** | | **Spring** | | **Autumn** | |
| --- | --- | --- | --- | --- | --- | --- | --- |
|  |  | **R** | ***p*-value** | **R** | ***p*-value** | **R** | ***p*-value** |
| Total FS17+Kalamata | Low vs High | **0.2872** | **0.0049** | N.S. | | N.S. | |
| FS17 |  | **0.3382** | **0.025** | n.d. | | n.d. | |
| Kalamata |  | 0.2296 | 0.0744 | n.d. | | n.d. | |
| Total High+Low | FS17 vs Kalamata | N.S. | | N.S. | | N.S. | |
| High |  | N.S. | | n.d. | | n.d. | |
| Low |  | N.S. | | n.d. | | n.d. | |
| Total FS17+Kalamata | Spring vs Autumn | **0.9007** | **0.0001** | / | / | / | / |
| FS17 |  | **0.9611** | **0.0021** | / | / | / | / |
| Kalamata |  | **0.8185** | **0.0019** | / | / | / | / |
