# Supplementary figures and images for: Differences in the Endophytic Microbiome of Olive Cultivars Infected by Xylella fastidiosa across Seasons
Source: Pathogens. 2020 Sep 2;9(9):723. doi: 10.3390/pathogens9090723 (PMC7558191; doi:10.3390/pathogens9090723)

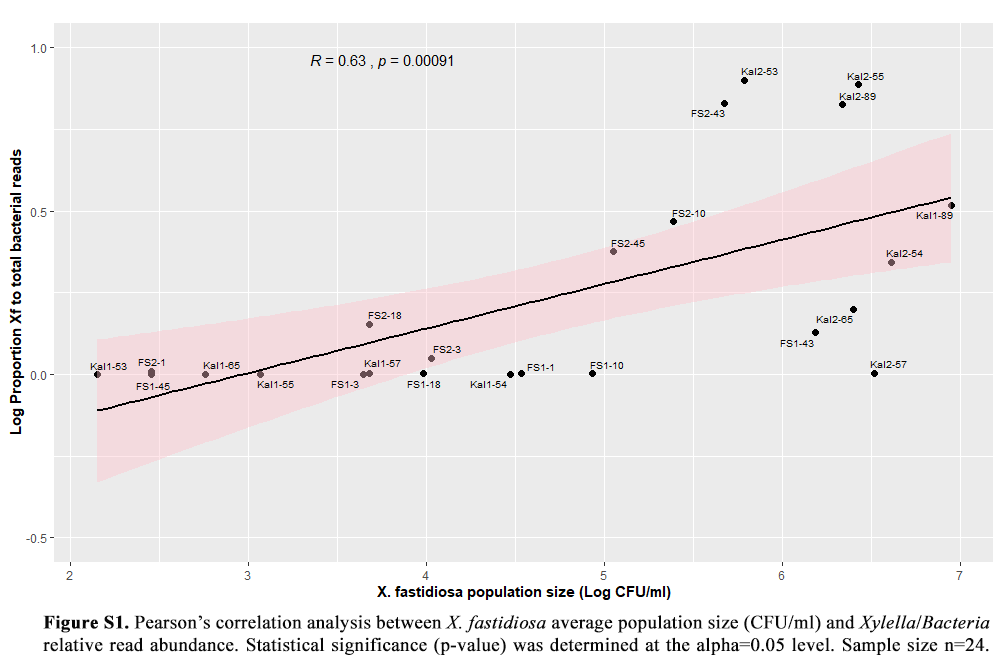

Supplement: Supplementary file 1 [file pathogens-09-00723-s001.zip › Figure S1.tif]

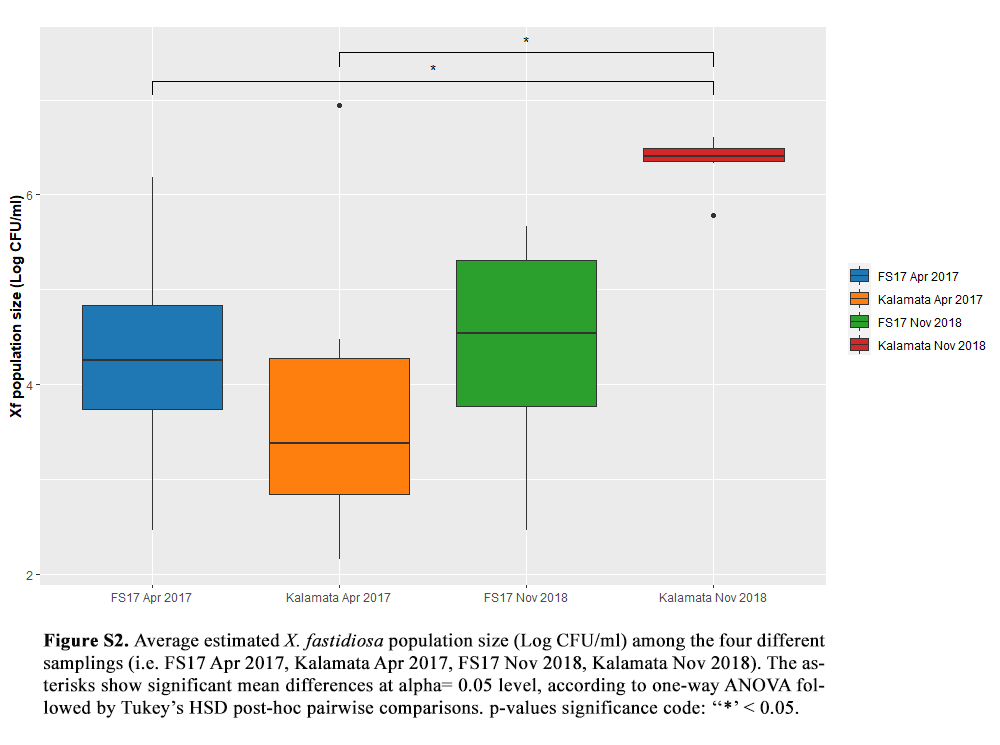

Supplement: Supplementary file 1 [file pathogens-09-00723-s001.zip › Figure S2.tif]

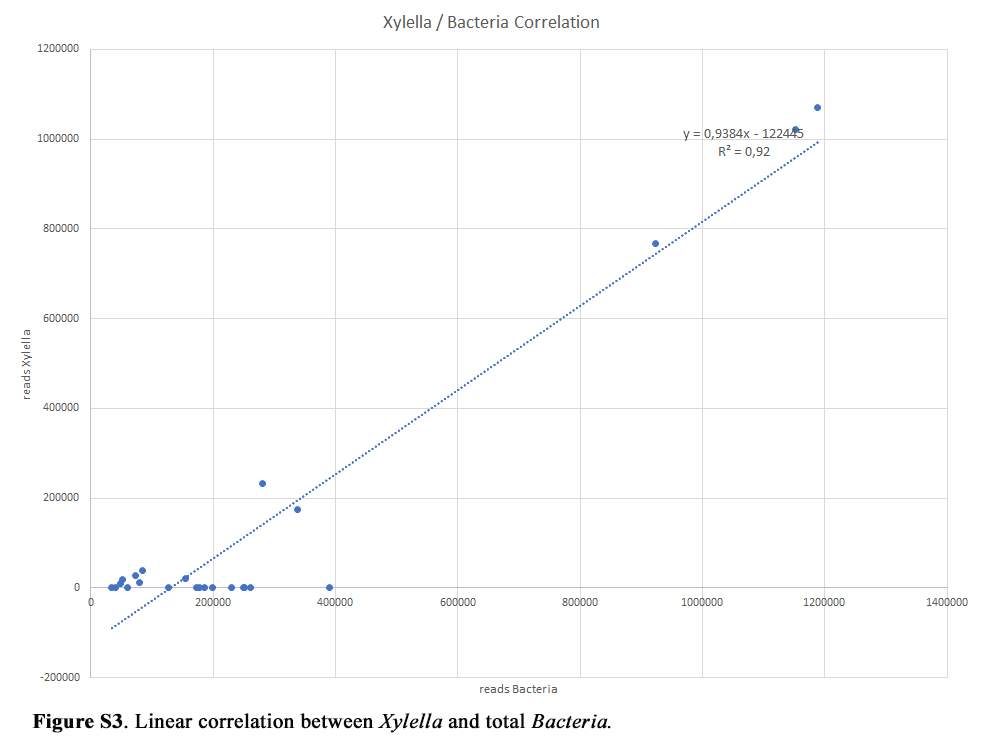

Supplement: Supplementary file 1 [file pathogens-09-00723-s001.zip › Figure S3.tif]
